# Cognitive therapy software for improving cognitive function for patients with mild cognitive impairment (SB‐DEX): A multicenter, prospective, comparative, randomized, independent rater‐blind, superiority, pivotal clinical trial to compare the safety and efficacy of the Cognitive therapy software ‘SUPERBRAIN DEX’ for improving cognitive function with a control group for patients with mild cognitive impairment (ClinicalTrials.gov ID NCT06264557)

**DOI:** 10.1002/alz70859_102394

**Published:** 2025-12-25

**Authors:** Hojin Choi, Yangki Minn, Sungmin Kang, Seunghyun Han

**Affiliations:** ^1^ Hanyang University Guri Hospital, Guri, gyeonggi‐do Korea, Republic of (South); ^2^ Kangnam Sacred Heart Hospital, Seoul, Seoul Korea, Republic of (South); ^3^ Rowan Corporation, Seoul, Seoul Korea, Republic of (South)

## Abstract

**Background:**

Cognitive intervention therapy has been proven to be a safe and effective technique for improving cognitive function in patients with mild cognitive impairment (MCI). Given the variety of intervention strategies within cognitive intervention therapy programs, the effectiveness and standardization of each program must be validated. The current computerized cognitive rehabilitation programs used in clinical practice are often foreign‐developed products that are difficult to apply directly to Korean elderly due to linguistic and cultural differences. Existing products developed in Korea tend to focus on gamified tasks that generate interest but have been reported to be weak in enhancing memory. Therefore, 'SuperBrain DEX'(developed by Rowan Co., Ltd.), a digital therapeutic device providing evidence‐based therapeutic intervention, has been developed to improve cognitive function in MCI patients. This clinical trial aims to assess the superiority of the cognitive function improvement effect of 'SuperBrain DEX' in patients with MCI compared to control groups and evaluate its safety for use.

**Method:**

The study targets MCI patients aged 50‐85 who meet the inclusion and exclusion criteria. Through 'SuperBrain DEX', cognitive training will be conducted for a total of 16 weeks, 7 times a week (each session lasting 15‐30 minutes). The program will assess the patient's cognitive training performance in real‐time and provide personalized training by applying individual cognitive function levels and basic information (gender, age, education, Z‐Score of cognitive tests) to artificial intelligence (AI). The control group will receive an educational booklet on lifestyle rules for dementia prevention. Cognitive function improvement will be confirmed through primary (K‐RBANS) and secondary (CDR, K‐MMSE‐2, K‐IADL, PRMQ, GDS‐15, QOL‐AD, ADAS‐Cog 14 tests) efficacy evaluations before and after the program.

**Result:**

The study is designed for 140 participants across 12 research institutions in Korea, with the first participant registered in January 2024. Currently, 140 participants (100%) are enrolled in the study.

**Conclusion:**

It is expected that the results will be reported at the time of the conference presentation.

